# A Low-Cost, Portable, Multi-Cancer Screening Device Based on a Ratio Fluorometry and Signal Correlation Technique

**DOI:** 10.3390/bios14100482

**Published:** 2024-10-07

**Authors:** Abdulaziz S. Alghamdi, Rabah W. Aldhaheri

**Affiliations:** Department of Electrical and Computer Engineering, King Abdulaziz University, Jeddah 21589, Saudi Arabia; aalghamdi3551@stu.kau.edu.sa

**Keywords:** cancer screening, porphyrins, ratio fluorometry, Walsh–Hadamard transform, lock-in amplifier

## Abstract

The autofluorescence of erythrocyte porphyrins has emerged as a potential method for multi-cancer early detection (MCED). With this method’s dependence on research-grade spectrofluorometers, significant improvements in instrumentation are necessary to translate its potential into clinical practice, as with any promising medical technology. To fill this gap, in this paper, we present an automated ratio porphyrin analyzer for cancer screening (ARPA-CS), a low-cost, portable, and automated instrument for MCED via the ratio fluorometry of porphyrins. The ARPA-CS aims to facilitate cancer screening in an inexpensive, rapid, non-invasive, and reasonably accurate manner for use in primary clinics or at point of care. To accomplish this, the ARPA-CS uses an ultraviolet-excited optical apparatus for ratio fluorometry that features two photodetectors for detection at 590 and 630 nm. Additionally, it incorporates a synchronous detector for the precision measurement of signals based on the Walsh-ordered Walsh–Hadamard transform (WHT)_w_ and circular shift. To estimate its single-photodetector capability, we established a linear calibration curve for the ARBA-CS exceeding four orders of magnitude with a linearity of up to 0.992 and a low detection limit of 0.296 µg/mL for riboflavin. The ARPA-CS also exhibited excellent repeatability (0.21%) and stability (0.60%). Moreover, the ratio fluorometry of three serially diluted standard solutions of riboflavin yielded a ratio of 0.4, which agrees with that expected based on the known emission spectra of riboflavin. Additionally, the ratio fluorometry of the porphyrin solution yielded a ratio of 49.82, which was ascribed to the predominant concentration of protoporphyrin IX in the brown eggshells, as confirmed in several studies. This study validates this instrument for the ratio fluorometry of porphyrins as a biomarker for MCED. Nevertheless, large and well-designed clinical trials are necessary to further elaborate more on this matter.

## 1. Introduction

In many regions of the world, the health and economic implications of cancer are substantial and growing. Cancer is a leading cause of global mortality, responsible for approximately 10 million deaths annually or one in six deaths worldwide [1]. Projections indicate a significant rise in the global cancer burden, with an estimated 30 million new cases expected by 2040, further straining health systems, people, and communities [2]. Furthermore, cancer has a significant impact on countries’ economic growth. One study estimated that the global economic burden of cancer will be USD 25.2 trillion (constant 2017 prices) between 2020 and 2050 [3]. In short, cancer’s rising incidence and high mortality coupled with a growing economic cost have created a worldwide burden that puts pressure on health systems and resources.

As a result, cancer screening tests have played an important role in the fight against cancer. Cancer screening identifies asymptomatic malignancies in a population to reduce advanced disease and allow for earlier treatment, thereby saving lives [4]. According to the United States Preventive Services Task Force (USPSTF), integrated screening programs saved 12.2–16.2 million life years between 1996 and 2020. This results in significant economic gains, with estimates ranging from USD 6.5 to 8.6 trillion based on current adherence rates [5]. Therefore, cancer screening tests are critical tools for cancer control.

Despite their benefits, existing screening tests have drawbacks. First, traditional screening tests, such as colonoscopy, mammography, endoscopy with biopsy, sigmoidoscopy, and low-dose computed tomography (CT) scans, may cause bleeding, perforation, discomfort, anxiety, radiation harm, or radiation-induced cancer [6,7,8,9,10,11,12,13,14,15,16,17]. Second, screening tests, such as prostate-specific antigen (PSA) and Pap smears, have a limited ability to discriminate between diseased and healthy individuals [18,19], leading to underdiagnosis or overdiagnosis. Third, the cost-effectiveness of some screening tests raises concerns within the healthcare community about their ability to deliver optimal value for allocated resources [20,21]. Fourth, limited access to expensive screening technologies, like magnetic resonance imaging, CT scans, and colonoscopies, for some cancers impedes timely diagnosis and worsens outcomes in low- and middle-income countries (LMICs) [22]. Therefore, a novel screening method that emphasizes early detection while putting the lowest burden on individuals and healthcare institutions is required.

As an alternative to traditional screening methods, the autofluorescence of red blood cells (RBCs) has been explored as a label-free approach for multi-cancer early detection (MCED), thus offering a minimally invasive and rapid screening tool at a significantly low cost [23,24,25,26,27,28,29,30,31,32,33]. Fluorescence spectroscopy analysis of fluorophores extracted from blood cells using acetone extraction revealed elevated porphyrin levels, with a characteristic increase in the peak at 630 nm compared to the peak at 590 nm, in patients with various cancers compared to healthy individuals and those with benign tumors [24]. These peaks at 590 and 630 nm are ascribed to basic and neutral porphyrins, respectively [24]. The ratio of fluorescence intensity at 630 and 590 nm shows promise for not only detecting malignant samples but also potentially indicating cancer stage, with a sensitivity ranging from 80% to 92% and a specificity between 78% and 100% depending on the cancer type and stage [23]. Moreover, in a study analyzing the ratio (630/590), healthy individuals exhibited an average ratio of approximately 0.5. In contrast, cancer patients demonstrated a progressive increase in this ratio, with early-stage cancer exhibiting values of approximately 0.8–1.0 and advanced-stage cancer exceeding 1.0 [24]. While this method is unable to identify the cancer type, it shows promise for cancer screening. Its label-free, non-invasive, rapid, and low-cost nature makes it a valuable tool.

Additionally, the selective accumulation of porphyrins in tumor tissue coupled with their enhanced fluorescence under acidic conditions make them promising candidates for cancer biomarkers. This is because the preferential uptake of porphyrins by tumor cells (due to some factors such as hypoxia, altered metabolism, and increased blood vessel permeability) leads to elevated levels of porphyrin in the bloodstream of patients with cancer [24]. The ability to selectively accumulate in tumor tissues has led to their potential use as sensitizers in a variety of medical applications, particularly in photodynamic therapy (PDT) [34]. Additionally, the excessive lactic acid produced by tumor tissue creates a more acidic microenvironment compared to normal tissue [24]. Consequently, porphyrins within the tumor microenvironment may experience protonation due to the altered pH conditions, which can enhance their fluorescence intensity [24]. Therefore, these properties have established porphyrins as promising cancer biomarkers.

On the other hand, this method provides substantial advantages compared to the common liquid biopsy-based MCED methods, such as circulating tumor cells (CTC) and cell-free DNA (cfDNA). First, MCED based on CTC or protein biomarkers typically requires labeling techniques for detection, which can be complex and costly [35]. In contrast, the label-free approach of the autofluorescence of porphyrin reduces the cost and complexity. Second, MCED methods based on genetic technologies require complex multi-step processes for sample preparation, which can be costly and time-consuming, and necessitate specialized laboratory facilities and specialized equipment [35]. By contrast, the autofluorescence of porphyrin method relies on an acetone extraction approach for sample preparation, which is a relatively simple process [24]. Third, some MCED methods rely on different analysis technologies, such as next-generation sequencing (NGS) and digital droplet PCR (ddPCR), which can be costly and complex [35]. On the contrary, fluorescence detection offers great advantages in terms of speed, cost, and operational or technical simplicity. Thus, this method offers significant advantages for cancer detection, including reduced turnaround time, low-cost screening, mass screening capabilities, and enhanced accessibility.

Additionally, integrating this method into a portable device offers distinct advantages compared to other portable cancer screening devices. For example, bio-affinity portable devices often depend on portable sensing assays using biosensors or electrochemical sensors supported by technologies such as nanotechnology and advancements in electrodes [36,37]. These sensors rely on different recognition methods, which can be complex, costly, and biomarker-dependent [36,37]. In contrast, autofluorescence is a direct and label-free approach, which is simple and cost-effective. In addition, smartphone-based systems employing imaging-based telemedicine or machine learning-assisted autofluorescence offer enhanced portability and accessibility but are currently limited to specific cancers, such as cervical and skin cancers [38,39,40,41,42,43,44]. In contrast, the detection of porphyrin, a molecule commonly found in tumors, holds promise for broader cancer screening applications. Similarly, wearable systems based on optical sensors and microwave imaging technologies offer great benefits in terms of remote monitoring and early detection but have limited accuracy and are limited to specific cancer types, such as breast and skin cancers [45]. In contrast, the autofluorescence approach offers greater accuracy and the potential for detecting multiple types of cancer. Overall, integrating this method into a portable device presents a compelling alternative to existing portable cancer screening devices.

Although the fluorescence method exhibits potential for cancer screening because of its advantages, significant improvements in instrumentation are necessary to translate its potential into real-world clinical practice, as with any promising medical technology. Its reliance on spectrofluorometry poses a challenge. Research-grade spectrofluorometers are sophisticated multi-purpose analytical instruments known for their technical complexity, high cost, and bulky form [46,47,48]. This limits their accessibility in non-research settings and necessitates training for operation, calibration, and data interpretation. In addition, their high cost can be a barrier in resource-constrained settings, and their operational complexity can hinder routine measurements outside of controlled laboratory environments [49,50,51]. These factors can limit their accessibility and cost-effectiveness for use in primary clinics or at the point of care for rapid and mass screening beyond research laboratories, especially in least developed countries (LDCs) or remote, underserved communities. Therefore, this necessitates the development of a low-cost, simple, automated, and portable instrument appropriately designed for routine cancer screening.

To address these limitations and unlock the full potential of the fluorescence method for cancer screening, we developed a novel, inexpensive, automated, and portable fluorometric device. This device incorporates the following: (1) an apparatus for ratio fluorometry at specific wavelengths (590 and 630 nm) using optical filters with a 405 nm light source and (2) a synchronous detector (built-in lock-in amplifier (LIA)) for the precise measurement of fluorescence signals based on Walsh-ordered Walsh–Hadamard transform (WHT)_w_ and circular shift. Although the concept and apparatus of ratio fluorometry are not new [52], they have not been exploited to develop an instrument for ratio fluorometry of porphyrins for cancer screening. In addition, while a few researchers have used the Walsh–Hadamard transform in fluorometry, none have perfectly adopted it as an LIA and, to some extent, as a tool for the easier identification of signals in the Walsh domain [53,54]. However, here, we exploit the Walsh-ordered Walsh–Hadamard transform’s time-variance property to develop a synchronous detector using a cyclic shift, similar to the phase shifter used in traditional LIAs. From an instrumentation perspective, an instrument based on a ratiometric fluorescence technique offers the following: (1) compensation for noise and light source fluctuations and (2) the direct measurement of the relative concentration within a sample without using calibration curves (i.e., no need to convert readings (voltage) to absolute concentration). Therefore, by combining these features, our device allows for a unique and promising approach to cancer detection, offering a more accessible, inexpensive, and user-friendly approach, making it suitable for deployment in various settings.

This paper is organized as follows: Section 2 details the steps followed in conducting this work, including the device’s design, fabrication, and evaluation; Section 3 presents the obtained results; and Section 4 highlights the findings and provides an interpretation.

## 2. Materials and Methods

Building on current spectrofluorometers and focusing on improving their limitations, the development and evaluation of a novel device, termed the automated ratio porphyrin analyzer for cancer screening (ARPA-CS), are detailed in this section. This device, which employs the ratio fluorometry of porphyrin derivatives as a biomarker for cancer screening, represents a refinement of the instrumentation for cancer screening via fluorescence method, aiming to transition it from research to practice.

### 2.1. ARPA-CS System Design

**Figure 1:**
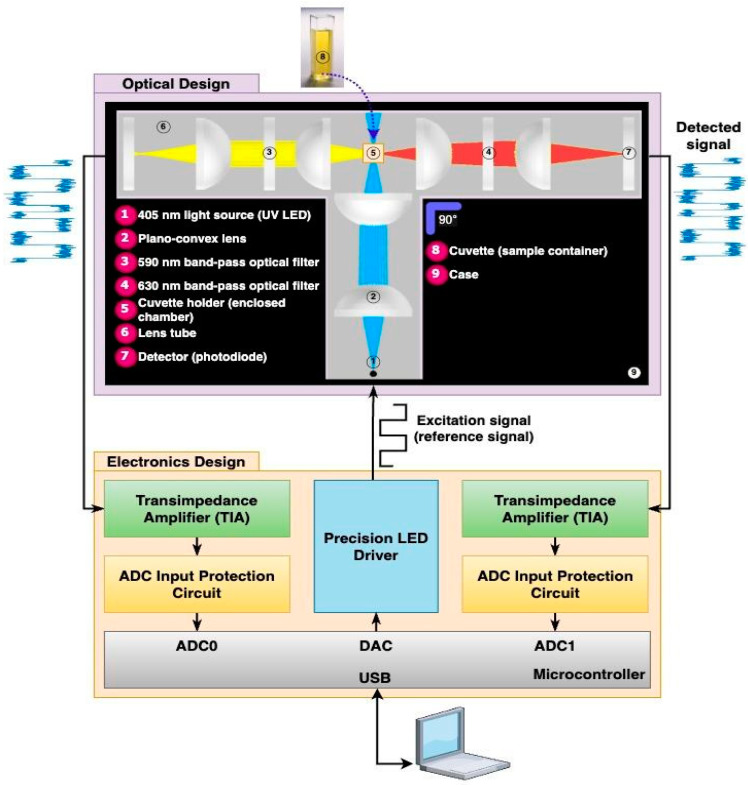
Schematic of the ARPA-CS design, including both the optical path and electronic design block diagram.

The ARPA-CS leverages fluorescence spectroscopy to determine the relative concentration of porphyrins within a sample, serving as a biomarker for cancer screening. The system function relies on measuring the ratio of two fluorescence signals emitted at specific wavelengths. To achieve this, the system design can be broadly categorized into two key aspects, as illustrated in Figure 1: (1) optical design and (2) electronic design.

The optical design determines how the light interacts with the sample and monitors the resulting fluorescence. The key elements of the optical design include the following: (1) a light source for sample excitation, (2) a sample chamber to hold the sample, and (3) two photodetectors optimized for 590 and 630 nm wavelengths for fluorescence detection. All of the components are arranged in a T-shaped configuration and housed in the upper compartment, as illustrated in Figure 1.

On the other hand, the electronic design functions as a digital synchronous detector (LIA). Its core components, as illustrated in Figure 1, comprise the following: (1) a microcontroller (to simultaneously transmit the modulation signal and receive the demodulated signal with its integrated digital-to-analog converter (DAC) and analog-to-digital converter (ADC), respectively), (2) a precision light-emitting diode (LED) driver for stable illumination, (3) a transimpedance amplifier (TIA) for signal conditioning, and (4) ADC protection circuits. The microcontroller (MCU) mounted on a printed circuit board (PCB) add-on module supports fluorescence measurement; it is housed in the lower compartment. The remaining components mounted on the PCBs are fitted within the optical system. Dedicated wires connect these components to the MCU’s add-on module.

**Figure 2:**
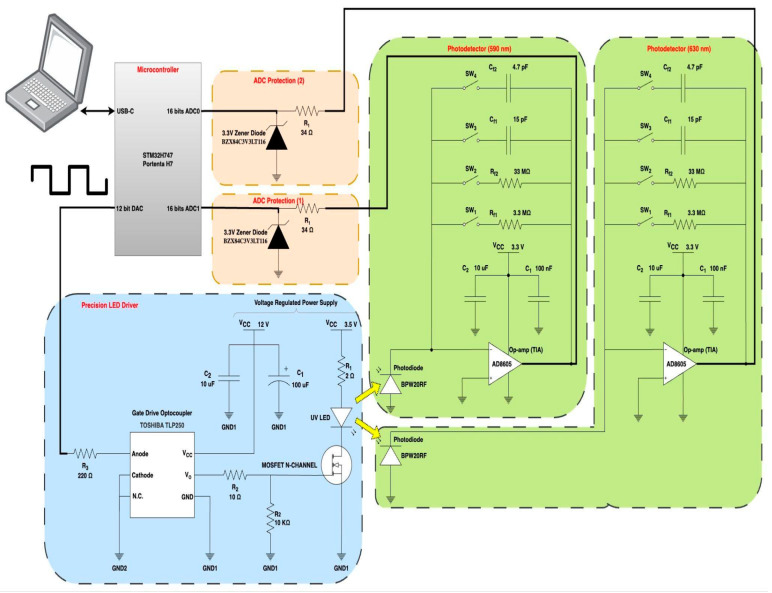
Schematic of the ARPA-CS optoelectronic circuit design.

Focusing on the specifics of the electronic design, Figure 2 illustrates the electronic circuitry, highlighting the design of two critical components. First, the adopted TIA design, as depicted in Figure 2, consists of an op-amp configured as an inverting amplifier and a feedback resistor (R_f_) in parallel with a feedback capacitor (C_f_). The configurable current-to-voltage gain is determined based on selected feedback resistance R_f_ and capacitance C_f_. The feedback capacitance is necessary to maintain stability. “Photodiode Amplifiers: OP Amp Solutions” by Jerald Graeme [55] was consulted for the design and noise analysis. Second, after considering different LED driver topologies, a low-side power-switching MOSFET transistor controlled by a gate driver (optocoupler) topology was adopted for the design because of its simplicity, low cost, and fast switching.

To elaborate on the system, we summarize the device’s operation as follows:

1. As shown in Figure 1, the process starts with the MCU producing the modulation signal. This signal comprises a train of square pulses (Walsh function) produced utilizing the MCU’s integrated 12-bit DAC.
2. The LED driver, providing amplitude modulation (AM), receives the modulation signal at its input, switching the high-power ultraviolet (UV) LED in an ON/OFF pattern (Walsh modulator).
3. Following the modulation, the light beam at 405 nm is directed through a collimator and then focused onto the sample within a cuvette placed in the sample chamber using two plano-convex lenses, exciting the sample, as shown in Figure 1.
4. As a result, the sample emits yellow and red lights (corresponding to 590 and 630 nm, respectively), pulsating in intensity at the same modulation frequency, with the intensity level reflecting porphyrin concentrations.
5. Two photodetectors capture the emitted light. As shown in Figure 1, each photodetector consists of a photodiode operated in photovoltaic mode, two plano-convex lenses, and a narrow bandpass filter. One detector’s filter is centered at 590 nm, while the other detector’s filter is centered at 630 nm. Thus, this configuration optimizes the system’s optical efficiency by maximally capturing the emitted light from the sample and directing it towards the photodiode.
6. Coupled with each photodiode on a PCB, a TIA converts the sample’s faint optical signal to a usable voltage range, and the signal is fed back to the microcontroller’s integrated 16-bit ADC for processing. To prevent potential damage from exceeding the ADC’s input voltage limit (3.3 V), a dedicated ADC protection circuit is employed to clip any incoming voltage that surpasses this threshold.
7. After detection, the microcontroller-based synchronous detector using signal processing techniques (covered in the following section) extracts the signal amplitude (of each photodetector) and rejects common noise that may arise from (1) internal electrical noise from the device, (2) electromagnetic interference from nearby sources, or (3) stray light leaking into the optical system.
8. Consequently, this signal processing enables the system to perform ratio fluorometry based on the ratio of the signal amplitudes at 630 nm and 590 nm. This ratio is indicative of porphyrin levels within the sample, thereby serving as a biomarker for cancer screening.

### 2.2. Synchronous Detector Based on (WHT)_w_ and Cyclic Shift

Influenced by the underlying principle of the phase-sensitive detection principle in the traditional LIA [56], we devised a synchronous detector based on the Walsh-ordered Walsh–Hadamard transform (WHT)_w_ and a cyclic shift. The essence of this technique was inspired by (1) Fourier filtering and (2) the lack of time-invariance properties of (WHT)_w_ [57].

A Fourier filter works by computing the Fourier transform of the signal, selectively truncating undesired frequencies (coefficients), and then performing the inverse Fourier transform to obtain the filtered signal, eventually boosting the signal’s signal-to-noise ratio (SNR) [58,59]. While this seems partially relevant to the goal of LIA (noise reduction), other Fourier-related orthogonal transforms suitable for pulsed applications like fluorescence measurements can be used (given the rich harmonic content of pulsed signals in the Fourier domain). Among these transforms is the (WHT)_w_, which can be adapted for quasi-lock-in detection. LIA based on (WHT)_w_ modulates the excitation signal using a Walsh function of known order (or sequency) and length. Then, this modulated signal excites a system under investigation. The LIA then receives the system’s response signal and transforms it into the Walsh spectrum to extract its features, mainly its strength or magnitude.

**Figure 3:**
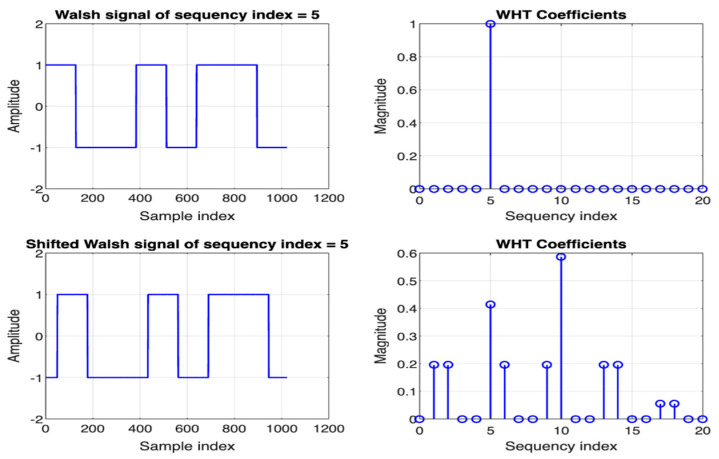
A Walsh function (**top left**) and its shifted version (**bottom left**). The corresponding sequency spectra of these signals are depicted on the right.

However, this technique alone is not sufficient to fulfill the functionality of traditional LIA because of the time-variance property of (WHT)_w_. This means any time delay or phase shift in the signal would change its features in the frequency (sequency) domain, as illustrated in Figure 3. Phase shift or time delay can arise from various factors, including the TIA’s feedback circuit and the system’s inherent response. Although the non-time-invariant property of (WHT)_w_ seems disadvantageous, it can be exploited to realize a real phase-sensitive detection technique.

**Figure 4:**
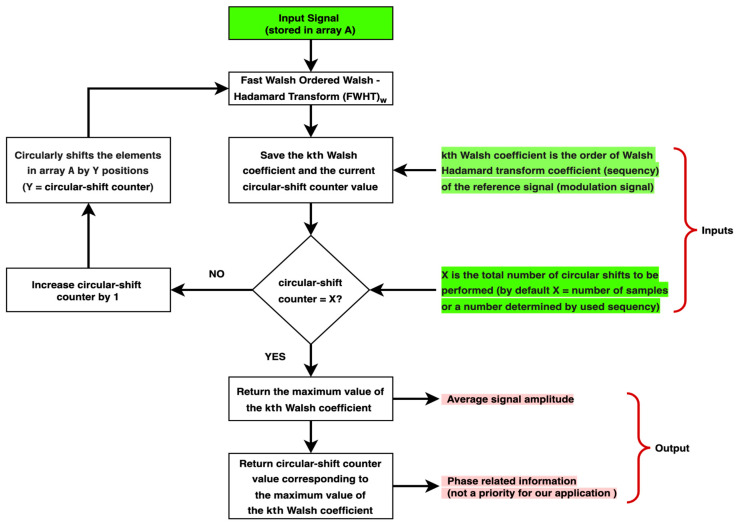
Algorithm for implementing an LIA using (FWHT)_w_ in conjunction with cyclic shift.

To achieve this, we need to compensate for the phase shift or time delay exerted on the signal to identify the best phase shift approximation that correlates to the maximum signal’s magnitude in the domain of (WHT)_w_. Given that (WHT)_w_ is time-variant to cyclic shift, by circularly shifting the input signal and monitoring the signal’s magnitude in the sequency domain, we can find the signal’s actual amplitude and phase (relative delay to the reference signal). This technique is similar to the phase-sensitive detection technique employed in traditional LIA. Figure 4 presents an algorithm that leverages the fast Walsh-ordered Walsh–Hadamard transform (FWHT)_w_ algorithm in conjunction with a cyclic shift to implement this technique.

To lessen the computation complexity of the algorithm, we reduced the number of required cyclic shifts. Instead of cyclically shifting the signal along its total length, we noticed that the required number of cyclic shifts varies depending on the signal’s Walsh ordering (number of zero crossings).

**Figure 5:**
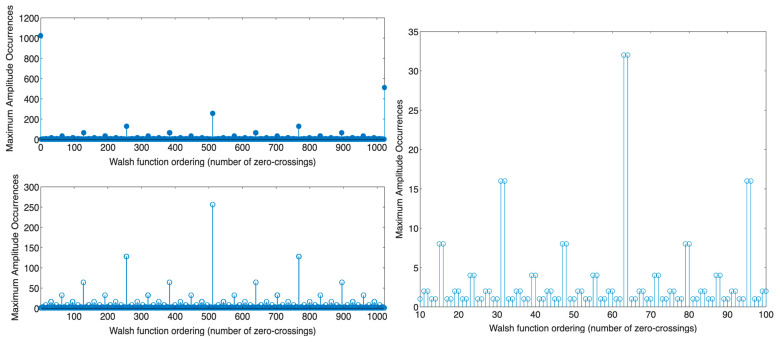
Distribution of maximum amplitude occurrences for Walsh functions for the order ranges of (**top left**) 0–1023, (**bottom left**) 1–1022, and (**right**) 10–100.

For all Walsh functions (order 0 to 1023) with lengths of 1024 points, we cyclically shifted each function 1024 times and applied (FWHT)_w_ after every single shift; then, we counted the times the function attained the maximum amplitude in the sequency domain. This total is shown for each Walsh function, as illustrated in Figure 5, which presents a pattern. We observed the following:

1. For Walsh functions of odd sequency (when (η+1)/2 or η/2 is an odd number, where the number of zero-crossings (η) is odd or even, respectively [60]), the total number of times the function attains maximum amplitude in the sequency domain is one.
2. For Walsh functions of even sequency (when (η+1)/2 or η/2 is an even number, where the number of zero-crossings (η) is odd or even, respectively [60]), the total number of times the function attains maximum amplitude in the sequency domain is more than one, defined as follows:
  - For the Walsh function of order N−1, where N is its length, the total number of times the function attains maximum amplitude in the sequency domain is N/2=1024/2=512 after cyclically shifting the signal along its entire length.
  - For the Walsh function of order (N−1)/2, the number of times the function attains maximum amplitude in the sequency domain is N/4=1024/4=256 after cyclically shifting the signal along its entire length.
  - For the remaining even Walsh functions, the total number of times the function attains maximum amplitude in the sequency domain may be 2, 4, 8, 16, 32, 64, or 128, determined by the function’s order.

Thus, odd Walsh functions are suitable for phase or time delay measurement, given that the number of times the function attains maximum (peak) amplitude in the sequency domain is one. Additionally, even Walsh functions are suitable for reducing the computational complexity of the algorithm to find the amplitude, given that the number of times the function attains maximum amplitude in the sequency domain is more than one (i.e., 2, 4, 8, 16, 32, 64, 128, 256, or 512).

**Figure 6:**
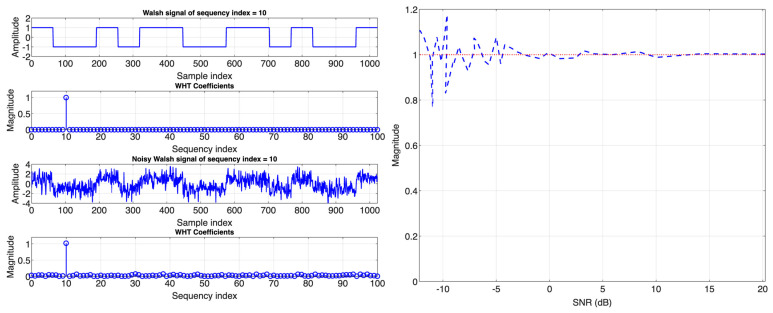
(**left**) Approximating amplitude of noisy Walsh signal using (WHT)_w_ and (**right**) performance of (WHT)_w_ against white noise (SNR: −12 dB to 20 dB).

To illustrate the effectiveness of this method against noise, Figure 6 (left) shows how (FWHT)_w_ can be used to approximate the amplitude of an excessively noisy Walsh signal (SNR = 1 dB). Figure 6 (right) shows the performance of this technique in estimating the amplitude of a Walsh signal contaminated by white noise with an SNR ratio between −12 dB and 20 dB.

To efficiently generate discrete Walsh functions of any order, we used a memory-efficient recursive algorithm detailed by M. I. Irshid in A Simple Recursive Definition for Walsh Functions [61], as it is much simpler and faster than other similar methods; it was eventually implemented in Python for this work. However, for implementing the (FWHT)_w_ algorithm, existing Python libraries lacked a suitable implementation for Walsh ordering. Thus, MATLAB’s fwht function was successfully ported to Python and used in this work.

### 2.3. Device Fabrication

**Figure 7:**
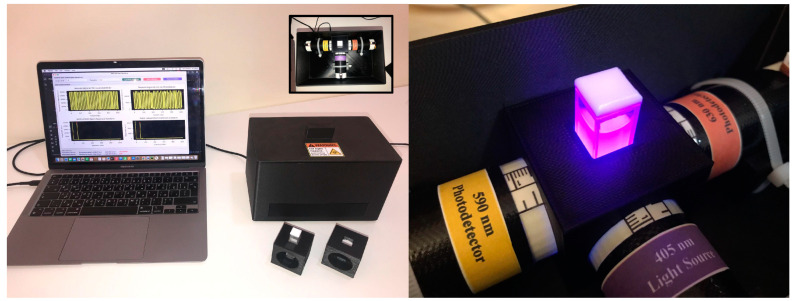
ARPA-CS photograph: (**left**) complete assembled device ready for operation and (**right**) sample chamber during irradiation.

The 3D-printed parts of the ARPA-CS device were first designed in 3D computer graphics software (Blender 4.2 LTS) and subsequently fabricated using fused deposition modeling (FDM) 3D printing with acrylonitrile styrene acrylate (ASA) filament, offering excellent resistance against UV radiation. On the other hand, the electronic circuit layouts were created using EasyEDA, a web-based electronic design automation (EDA) tool, while fabrication and assembly of the PCBs were completed by JLCPCB. The system’s estimated cost is USD 1045, with optics adding up to USD 842. Figure 7 shows a photograph of the complete assembled system.

### 2.4. Modulation Frequency Selection

Photodetection systems are susceptible to at least three types of noise: thermal (Johnson) noise, shot noise, and 1/f (pink) noise [62]. While 1/f noise is frequency-dependent, both Johnson noise and shot noise are frequency-independent and inevitable in measurement [62]. To mitigate the impact of 1/f noise and improve the SNR, a modulation frequency of approximately 500 Hz, which is equivalent to a Walsh function of order 1023 (number of zero-crossings) with 1 ms sampling intervals, was selected. However, selecting the optimal frequency necessitates a careful balance between minimizing 1/f noise and (1) potential interference from ambient sources or (2) limitations within the system, such as the operational amplifier’s (op-amp) gain–bandwidth product (GBWP) and the photodetector’s cutoff frequency. Thus, the optimal modulation frequency should yield the highest achievable SNR.

### 2.5. ARPA-CS Signal Processing

The system utilized a 1024-point Walsh function of order 1023 (number of zero-crossings) with 1 ms intervals to modulate the UV LED. The resulting fluorescence signals were captured by the two photodetectors (590 and 630 nm) at a sampling rate of approximately 1 kHz using the MCU’s built-in 16-bit ADCs. The user interface displayed the phase-adjusted (correlated) fluorescence data alongside its (WHT)_w_ for visualization. Additionally, the user interface results section presented the fluorescence intensities from both detectors and the ratio of the intensities at 630 and 590 nm.

### 2.6. Software Implementation and Graphical User Interface (GUI)

Synchronous detection (lock-in detection) was implemented completely in the software. The ARM Cortex M4/M7 microcontroller (Portenta H7) mounted on the add-on module facilitated the lock-in detection, which was achieved by utilizing the microcontroller’s built-in DAC to generate the reference signal (Walsh modulation signal) and its built-in ADC to capture the fluorescence signals (demodulated Walsh signal).

**Figure 8:**
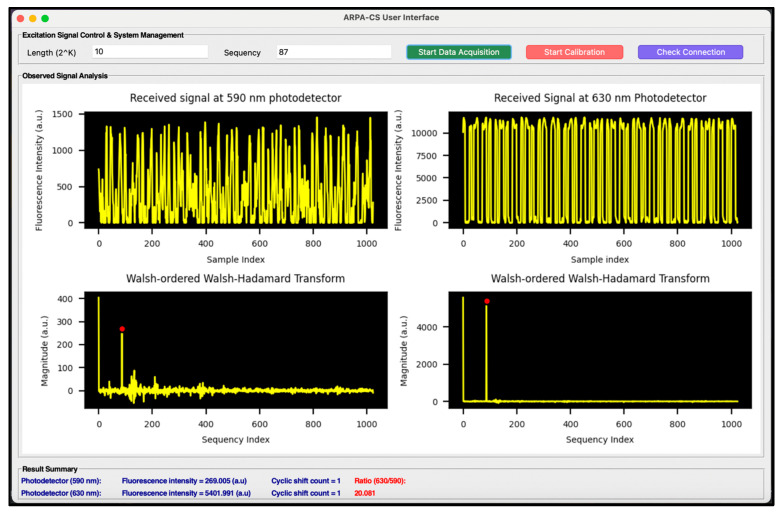
ARPA-CS graphical user interface.

For user interaction and data visualization, a graphical user interface was developed using Python Tkinter library, as shown in Figure 8. This dashboard application facilitated communication with the microcontroller via a USB serial port, enabling users to visualize results and control excitation (reference) signals.

### 2.7. Device Calibration

Despite the identical design of the photodetectors, potential manufacturing variations, temperature, stray light leaks, and electronic noise can cause offsets between photodetectors. To account for this error, a blank solution was employed to establish a zero baseline for subsequent measurements. The measured fluorescence from the blank solution was subtracted from subsequent measurements with actual samples to compensate for this offset.

### 2.8. Evaluation Method

To assess the device for its intended application, we evaluated four aspects: (1) device performance specifications (e.g., limit of detection (LOD), dynamic range), (2) ratio fluorometry, (3) simulant test, and (4) synchronous detection efficiency.

#### 2.8.1. Device Performance Specifications

The device’s ability to measure fluorescence at two separate emission wavelengths allows for its performance to be directly assessed as a standard single detector fluorometer. This assessment is possible because the detectors themselves are nearly identical, with the only difference being the wavelength of light each detector is designed to capture. Key specifications commonly used for performance validation of analytical instruments were considered [63]. A comprehensive overview of these specifications is provided in Table 1.

To realize these specifications, a custom standard solution designed to replicate our target molecules (porphyrins) is ideal. However, such a solution is expensive and time-consuming to synthesize. Therefore, given that our primary objective is ratio fluorometry, a substitute material exhibiting the following properties was sought: (1) similar excitation and emission spectra (excitation approximately 405 nm and emission peaks at 590 and 630 nm) and (2) a low quantum yield, analogous to porphyrins.

**Figure 9:**
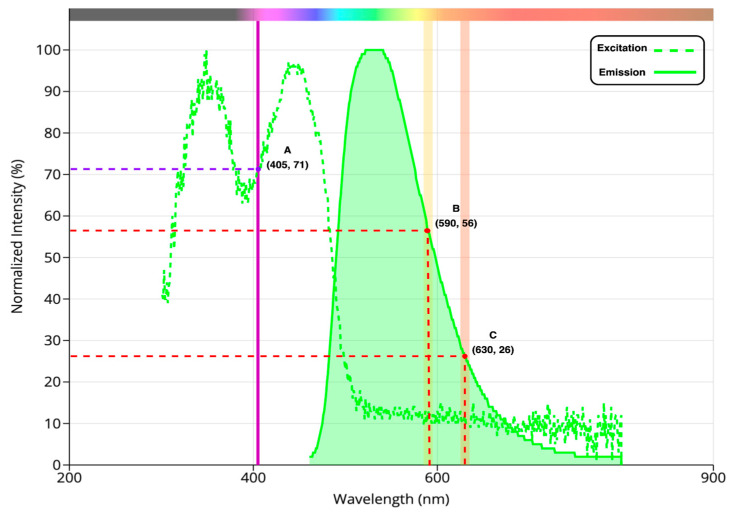
Excitation and emission spectra of riboflavin. Point A on the spectrum indicates the excitation wavelength, while points B and C represent emission wavelengths.

Riboflavin (vitamin B2) fulfills the requirements. Its broad emission covers our target range, and it is readily available with a suitable quantum yield (approximately 0.3). Figure 9 [64] shows the excitation and emission spectra of riboflavin.

#### 2.8.2. Ratio Fluorometry

Ratio fluorometry performance was tested via verification with a reference standard. Since riboflavin has well-known emission and excitation spectra, it was utilized as a standard solution. As observed in Figure 9, the ratio of fluorescence at 630 nm and 590 nm is predicted to be roughly 0.4. The ratio fluorometry of serially diluted solutions of known concentrations of riboflavin was measured for the assessment. The accuracy of the measured ratios was then verified by comparing them to the predicted value of 0.4.

#### 2.8.3. Simulant Test

Clinical testing aimed to validate the ARPA-CS’s performance for cancer screening using actual blood samples. Real blood samples are ideal for real-world testing, but acquiring them was not possible. On the other hand, porphyrin derivatives could be easily obtained by extracting them from easily accessible sources, like brown eggshells [65,66].

Protoporphyrin IX (classified as a porphyrin) is the main component of brown eggshell pigment, with other porphyrin derivatives present in trace amounts [67]. Therefore, brown eggshells were used as a substitute source of blood samples, which were extracted using the technique outlined below. However, it was predicted that the ratio fluorometry (630/590) would be greater by orders of magnitude because protoporphyrin IX has an emission peak at approximately 630 nm and an excitation maximum of approximately 400 nm [68]. In short, this approach made it easier to validate the device’s performance.

#### 2.8.4. Synchronous Detection Efficiency

To validate the efficiency of our technique, we needed to demonstrate how the applied modulation positively influenced the signal-to-blank ratio (SBR). A linear regression analysis of SBR as a function of Walsh modulation’s sequency index was used to assess the strength of the relationship.

To assess these aspects, the evaluation employed the following materials:

(a) A stock solution of 0.3 mg of riboflavin dissolved in 100 mL distilled water, making the concentration 3 μg/mL or 3 ppm.
(b) A porphyrin solution was prepared by treating crushed brown eggshells with a 2 M HCl–ethyl acetate mixture in a flask. After stirring at approximately 22 °C for 10–20 min, the organic layer was isolated via filtration [65].

## 3. Results

Building upon the evaluation method described in the previous section, this section presents the findings of the four-part evaluation method. Measurements were conducted at room temperature using a 3500 μL cuvette containing a sample volume of approximately 3 mL.

### 3.1. Device Performance Specifications

**Figure 10:**
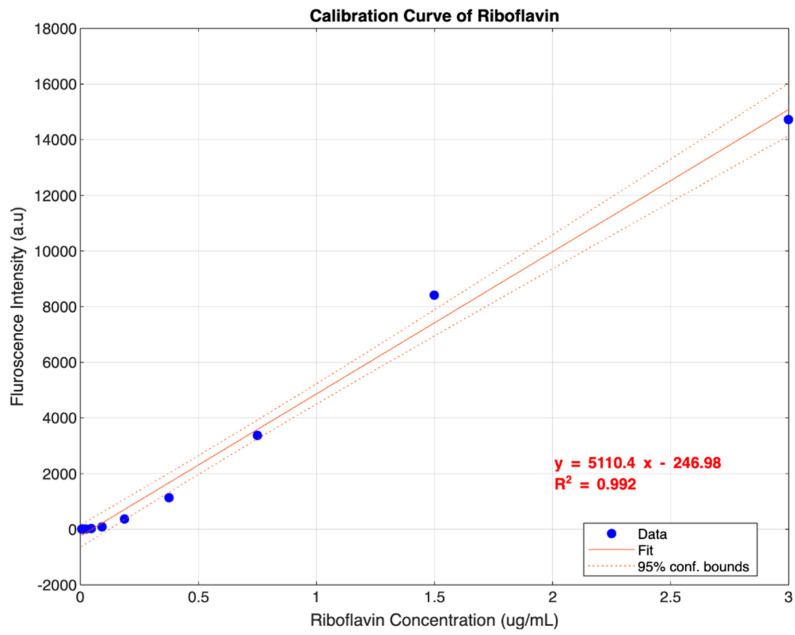
Calibration curve of riboflavin measured at 590 nm, based on data from 10 samples.

Following the key specifications outlined in Table 1, we generated a calibration curve (Figure 10) of a single detector (590 nm) by measuring the fluorescence intensity of serially diluted standard solutions of riboflavin diluted by a factor of two (10 solutions including the blank). This curve was then used to find the values of the specifications, as shown in Table 2. The resulting data, including calibration curve, repeatability, and stability data, are presented in Table 3, Table 4, and Table 5, respectively.

### 3.2. Ratio Fluorometry

Ratio fluorometry measurements were taken on three serially diluted riboflavin standard solutions of known concentrations. The concentrations of the standard solutions and their corresponding fluorescence intensities and ratios are presented in Table 6. The measurements presented are the average values of four measurements.

### 3.3. Simulant Test

A porphyrin solution from brown eggshells was analyzed using ratio fluorometry. Table 7 presents the fluorescence data and the corresponding ratios. The measurements presented are the average values of four measurements.

### 3.4. Synchronous Detection Efficiency

**Figure 11:**
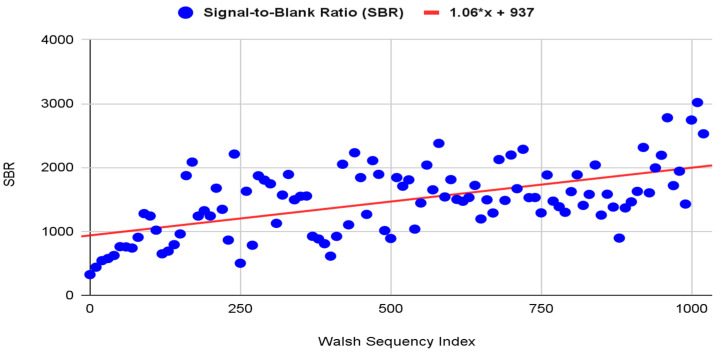
Linear regression analysis of SBR as a function of the Walsh sequency index.

The relationship between the signal-to-blank ratio (SBR) and the Walsh modulation’s sequency index was investigated using linear regression analysis (Figure 11).

## 4. Discussion

As shown in the calibration curve (Figure 10) and specifications (Table 5), the device exhibits a low LOD, a wide dynamic range, linearity, repeatability, stability, and portability. First, an LOD of 0.296 µg/mL indicates high sensitivity for detecting low quantities of fluorescent molecules and falls below the typical concentrations of fluorescent molecules found in several biological samples. For example, riboflavin concentrations in RBCs and urine are 0.5 and 0.8 µg/mL, respectively [69]. Second, the dynamic range of more than four orders of magnitude is excellent, given that many commercially available research-grade instruments offer a range of three to five orders of magnitude [70,71], allowing for the measurement of a wide range of concentrations without adjustment. Third, a linearity of 0.992 indicates a strong correlation between the measured fluorescence intensity and the actual analyte concentration, close to the ideal value of 1. Fourth, a repeatability of 0.21% indicates high precision, meaning the device produces consistent results with minimal variation between repeated measurements. Additionally, a stability of 0.60% indicates consistent results over a long timeframe or across measurement sessions. Fifth, a device volume of 3150 cm^3^ and a weight of 740 g allow for easy portability compared to many commercially available research-grade instruments, which are bulky and heavy [69,70]. Overall, this device’s high sensitivity, wide range, and portability make it suitable for fluorescence measurements.

On the other hand, examining the ratio fluorometry measurements (shown in Table 6) is important for verifying device function. The ratio of the intensities (*I*_630nm_/*I*_590nm_) highlighted in Figure 9 indicates ratio fluorometry accuracy. Because of the high linearity that photodiodes exhibit [55,62], a ratio of 0.4 is expected. The ratio holds steady even at different concentrations, indicating that the measurements are accurate.

Additionally, the ratio fluorometry measurement of the porphyrin solution, as shown in Table 7, is valid and expected. A ratio of 49.82 (approximately 50:1) was ascribed to the predominant concentration of protoporphyrin IX (PPIX) in the brown eggshells, with other porphyrins present in trace amounts [54]. This alignment between the measured ratio and the expected value based on PPIX concentration strengthens the validity of the ratio fluorometry measurements.

Shifting the focus to the synchronous detection efficiency, the SBR tends to rise with Walsh modulation’s sequency index increases, as shown in Figure 11. Based on the regression analysis, the SBR doubles as the sequency index of the Walsh modulation signal increases from 0 to 1023. The low SBR at low frequencies can be attributed to the 1/f noise (pink noise), which increases in power as the signal frequency decreases. In addition, the variation in SBR is expected given the stochastic nature of noise. Therefore, synchronous detection based on (WHT)_w_ and cyclic shift proved to be effective for boosting the SBR.

These findings pave the way for future clinical trials. Blood samples from healthy volunteers and patients with known stages and etiology are beneficial for further investigation.

While ARBA-CS demonstrates considerable promise, its current limitations should be acknowledged in the future. For instance, ARBA-CS supports single sample analysis by analyzing one sample at a time in a cuvette, which is a time-consuming and slow process for a large number of samples given the required manual handling of samples. For high-throughput analysis, ARBA-CS can be optimized for measuring fluorescence in microplates, allowing for rapid analysis of multiple samples simultaneously, making it suited for mass screening. Each well in the microplate would ideally be equipped with a dedicated emitter/detector set for optimal performance.

In addition, a primary obstacle to the future adoption of this technology into mainstream clinical practice is the necessity for rigorous clinical validation and regulatory approval. Large-scale trials are necessary to establish the baseline real-world sensitivity, specificity, and accuracy of this technology, as stated by several studies [24,26,31,32]. The outcome of these trials would determine its integration into existing cancer screening programs and probably its use as a standard or complementary method to conventional methods such as tissue biopsy or its use in cancer monitoring and post-treatment surveillance. Moreover, obtaining regulatory approval from agencies like the FDA or equivalent bodies can be a time-consuming and complex process. Nevertheless, its potential benefits outweigh its challenges, making it an asset in advancing healthcare.

## 5. Conclusions

The development of a fluorometric instrument designed for multi-cancer screening via the ratio fluorometry of porphyrins is presented in this study. An evaluation of key specifications commonly used for the performance validation of analytical instruments indicates the instrument’s capability for fluorescence measurements, given its high sensitivity, wide range, and consistent results (repeatability and stability). Additionally, its low cost and portability (low weight and small volume) make it suitable for use in different settings, particularly primary clinics. Consistent ratio measurements, whether for standard riboflavin or porphyrin solutions, validate the device’s ratio fluorometry measurements. Similarly, the increased SBR confirms the effectiveness of synchronous detection for improving signal quality. This study provides initial evidence for the validity of this instrument for the ratio fluorometry of porphyrins, a potential biomarker for MCED. However, large and well-designed clinical trials are required to provide more information on this subject.

## Figures and Tables

**Table 1:** Performance specifications.

Specification	Description	Method
LOD	Minimum detectable concentration of a specific molecule.	Analyze the calibration curve using linear regression (fluorescence intensity vs. analyte concentration). Limit of Detection=3.3×σ/S where σ is the standard deviation of the response and S is the slope of the calibration curve.
Dynamic Range	The interval between the upper and lower concentrations of the analyte (including these concentrations) for which a suitable level of linearity has been demonstrated.	Analyze visually from the calibration curve.
Linearity	The ability to produce a proportional relationship between the concentration of the fluorescent molecule and the measured fluorescence intensity.	$R^{2}$ in regression analysis is the measure of linearity strength between concentration and fluorescence intensity.
Repeatability	The ability to produce consistent fluorescence readings for the same sample under identical conditions. Relative standard deviation (RSD) is used to express repeatability.	Measure the fluorescence intensity of a sample 10 times in a short time frame (1 min interval) under identical conditions. Calculate sample standard deviation, s RSD=s/Mean×100 A low RSD suggests good repeatability.
Stability	Consistency in producing the same values over time for the same sample. RSD is used to express stability.	Measure the fluorescence intensity of a sample solution 10 times over a long period of time (1 day interval). Calculate sample standard deviation s RSD=s/Mean×100 A low RSD suggests good stability.

**Table 2:** Determined performance specifications.

Specification	Value	Comment
LOD	0.296 µg/mL or 296 ppb	σ = 459
Dynamic Range	More than four orders of magnitude	—
Linearity	0.992	—
Repeatability	0.21%	—
Stability	0.60%	—
Volume	$3150\;{cm}^{3}$	—
Weight	740 g	—

**Table 3:** Calibration curve data.

Concentration (µg/mL) or (ppm)	Fluorescence Intensity (590 nm)	Calibrated Readings	Modulation Sequence
3	14,745.287	14,727.813	1023
1.5	8432.916	8415.169	
0.75	3393.295	3375.548	
0.375	1153.053	1135.306	
0.187	386.782	369.035	
0.093	109.650	91.903	
0.046	38.566	20.819	
0.023	20.458	2.711	
0.011	18.757	1.01	
Blank solution	17.474	0	

**Table 6:** Data from ratio fluorometry of riboflavin standard solutions.

Analyte	Concentration (µg/mL)	Calibrated Fluorescence Intensity (590 nm)	Calibrated Fluorescence Intensity (630 nm)	Ratio (*I*_630nm_/*I*_590nm_)
Riboflavin	3	14,727.813	6274.461	0.426
	1.5	8415.169	3559.262	0.422
	0.75	3375.548	1358.86	0.402

**Table 7:** Data from ratio fluorometry of porphyrin solution.

Analyte	Calibrated Fluorescence Intensity (590 nm)	Calibrated Fluorescence Intensity (630 nm)	Ratio (*I*_630nm/_*I*_590nm_)
Porphyrin	96.455	4805.743	49.82

**Table 4:** Repeatability data.

Reading No.	Fluorescence Intensity at 590 nm (Riboflavin Sample)	Modulation Sequency
1	21,161.072	1023
2	21,114.001	
3	21,167.485	
4	21,207.688	
5	21,118.311	
6	21,228.713	
7	21,160.219	
8	21,241.393	
9	21,195.318	
10	21,222.090	

**Table 5:** Stability data.

Reading No.	Fluorescence Intensity at 590 nm (Riboflavin Sample)	Modulation Sequency
1	21,740.362	1023
2	21,823.546	
3	21,987.590	
4	21,966.765	
5	21,982.763	
6	21,998.738	
7	21,916.649	
8	22,119.437	
9	22,147.506	
10	22,134.718	

## Data Availability

Data are contained within the article.
